# Massive right coronary air embolism in the right coronary artery during left coronary angiography: A case report

**DOI:** 10.3892/etm.2013.939

**Published:** 2013-01-30

**Authors:** CHANG-BUM PARK, HUI-JEONG HWANG, JIN-MAN CHO, BYUNG-HYUN JO, CHONG-JIN KIM

**Affiliations:** Department of Internal Medicine, Kyung Hee University, Kyung Hee University Hospital at Gangdong, Seoul 134-727, Republic of Korea

**Keywords:** air embolism

## Abstract

Coronary air embolism is one of the inadvertent complications of coronary angiography. We report a case of unexpected massive right coronary air embolism during left coronary angiography with a JL4 diagnostic catheter. This report demonstrates that air embolism may occur in the contralateral coronary artery and therefore complete air aspiration must be ensured during coronary angiography.

## Introduction

Coronary air embolism is a well-recognized complication of coronary catheterization and occurs in 0.1–0.3% of cases (1,2). The causes of coronary artery air embolism include incomplete aspiration of angiographic or guiding catheters, balloon rupture, insinuation of air with balloon catheter introduction or withdrawal, structural failures of the equipment and constant negative suction of self-venting catheters left out of the body (2,3). Herein, we report a rare case of unexpected contralateral coronary artery air embolism during left-side coronary angiography.

## Case report

**Figure 1:**
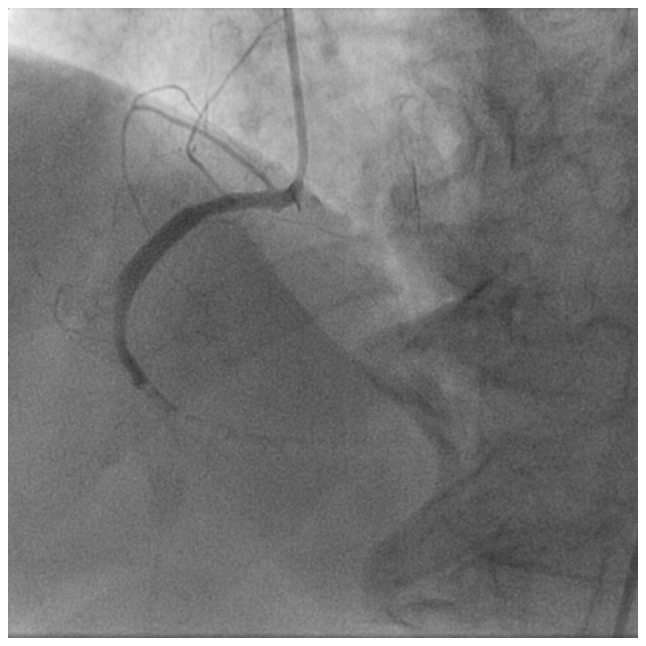
Right coronary artery immediately after left coronary angiography. A massive air embolism interrupted the blood flow in the mid-portion of the right coronary artery.

**Figure 2:**
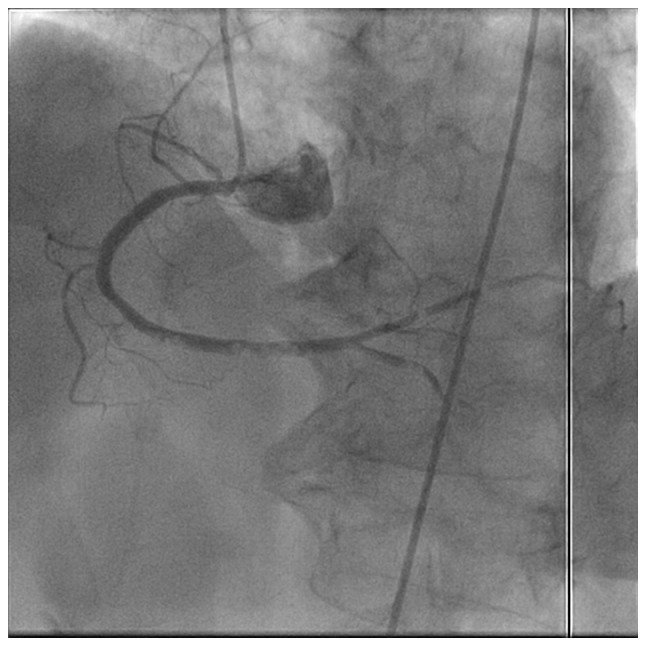
Right coronary angiogram immediately after left coronary angiography. Small shadow defects were considered to be air bubbles present in the distal portion of the right coronary artery and later the bubbles dispersed distally.

A 65-year-old female with stable angina was scheduled to undergo follow-up for coronary angiography 9 months after the performance of an index procedure. According to the routine procedure, a standard JL4 diagnostic catheter via the right femoral artery was employed to catheterize the left coronary artery. On engagement and initial injection of the left coronary artery, the patient began to experience severe chest pain and the ST segment in leads II and III began to rise. Immediately, the catheter was exchanged and right coronary artery angiography was performed. Multiple small bead-like shadow defects suspected to be air bubbles were present in the mid-portion of the right coronary artery and interruption of blood flow was observed (Fig. 1). The patient went into a shock state with complete atrioventricular block and hypotension. Immediately, the patient received 100% oxygen inhalation through a facemask. Contrast dye was forcefully injected several times and the air bubbles dispersed distally (Fig. 2). The treatments were successful in terms of improvement in the condition of the patient. Subsequently, the patient recovered from the state of shock. Later, angiography confirmed the disappearance of air embolism and sufficient blood flow was obtained in the right coronary artery. The patient had an uneventful overnight stay and was discharged the following day with a normal electrocardiogram (ECG) and cardiac enzyme levels. Written informed consent was obtained from the patient.

## Discussion

Our case demonstrated an unusual process by which air was introduced into the right coronary artery during left coronary artery angiography. The cause may be due to incomplete aspiration of the angiographic catheter. However, the mechanism of contralateral air embolism is unclear. Inoue *et al* (4) reported similar cases and insisted that the air was injected through the sidehole. However, we used a diagnostic catheter without a sidehole so this does not explain the mechanism of contralateral air embolism. Another report (5) speculated that the air trapped in the catheter lumen followed the path of least resistance and exited forcefully from the end hole of the diagnostic catheter. The force of the contrast injection directed the mixture of blood-air towards the medial wall of the left aortic cusp from where it rebound towards the right coronary cusp, resulting in its admixture with the forward ejectate, which was then distributed into the right coronary artery and aortic arch vessels.

Immediate management of coronary air embolism has not yet been established. When small to moderate amounts of air are involved, inhalation of 100% oxygen is appropriate since oxygen facilitates the absorption of the air embolus through the microcirculation. When large amounts of air are involved, more aggressive modalities, including air aspiration and forceful injection of saline or contrast medium are suggested (2,3,6,7). Previously, stirring the mass of air using a 0.014-inch guidewire and balloon catheter for coronary angioplasty has also been suggested, which worked by breaking up the larger air mass into a number of smaller bubbles to increase the surface area (4).

Prevention of iatrogenic massive air embolism is paramount. Back bleeding from the catheter during its introduction is protective against the entrapment of any air present in the catheter. In addition, the catheter should be tested in the ascending aorta, distal to the aortic cusp to prevent the occurrence of adverse events (5).

In conclusion, awareness that air embolism may occur in contralateral coronary artery angiography is essential, and complete air aspiration during coronary angiography should be ensured.
